# Division of developmental phases of freshwater leech *Whitmania pigra* and key genes related to neurogenesis revealed by whole genome and transcriptome analysis

**DOI:** 10.1186/s12864-023-09286-5

**Published:** 2023-04-17

**Authors:** Jiali Liu, Jinxin Liu, Mingyue Li, Lisi Zhou, Weijun Kong, Hailin Zhang, Panpan Jin, Fuhua Lu, Gufa Lin, Linchun Shi

**Affiliations:** 1 Key Lab of Chinese Medicine Resources Conservation, State Administration of Traditional Chinese Medicine of the People’s Republic of China, Institute of Medicinal Plant Development, Chinese Academy of Medical Sciences & Peking Union Medical College Beijing 100193, China Engineering Research Center of Chinese Medicine Resource, Ministry of Education, Beijing, 100193 China; 2 Renji Hospital, Shanghai Jiao Tong University School of Medicine, Shanghai, 200127 China; 3 School of Traditional Chinese Medicine, Capital Medical University, Beijing, 100069 China; 4 Key Laboratory of Spine and Spinal Cord Injury Repair and Regeneration of Ministry of Education, Orthopaedic Department of Tongji Hospital, School of Medicine, School of Life Sciences and Technology, Tongji University, Shanghai, 200065 China

**Keywords:** *Whitmania pigra* Whitman, Transcriptional dynamics, Morphogenesis, Signal pathways, Neurogenesis

## Abstract

The freshwater leech *Whitmania pigra* (*W. pigra*) Whitman (Annelida phylum) is a model organism for neurodevelopmental studies. However, molecular biology research on its embryonic development is still scarce. Here, we described a series of developmental stages of the *W. pigra* embryos and defined five broad stages of embryogenesis: cleavage stages, blastocyst stage, gastrula stage, organogenesis and refinement, juvenile. We obtained a total of 239.64 Gb transcriptome data of eight representative developmental phases of embryos (from blastocyst stage to maturity), which was then assembled into 21,482 unigenes according to our reference genome sequenced by single-molecule real-time (SMRT) long-read sequencing. We found 3114 genes differentially expressed during the eight phases with phase-specific expression pattern. Using a comprehensive transcriptome dataset, we demonstrated that 57, 49 and 77 DEGs were respectively related to morphogenesis, signal pathways and neurogenesis. 49 DEGs related to signal pathways included 30 *wnt* genes, 14 *notch* genes, and 5 *hedgehog* genes. In particular, we found a cluster consisting of 7 genes related to signal pathways as well as synapses, which were essential for regulating embryonic development. Eight genes cooperatively participated in regulating neurogenesis. Our results reveal the whole picture of *W. pigra* development mechanism from the perspective of transcriptome and provide new clues for organogenesis and neurodevelopmental studies of Annelida species.

**Supplementary Information:**

The online version contains supplementary material available at 10.1186/s12864-023-09286-5.

## Background

Leech species are members of the Annelida phylum, widely distributed in reservoirs, lakes, streams, and even oceans all over the world. They can survive in a wide range of temperatures, humidity, salinity, and water pressures [1]. However, leech species are at the forefront of aquatic organisms threatened by human activities [2]. In addition of being overused for medicinal purpose [3, 4], leeches are challenged by environmental changes, including widely used pesticides [5], and the impact of urbanization [6]. Dense residential areas, concentrated energy consumption and fugitive emissions have led to sustained and increased emissions of polycyclic aromatic hydrocarbons and heavy metals (especially lead) [7], seriously damaging the water environment and soil environment where leeches live and breed. The presence of specific leech species is often regarded as an indicator of the aquatic environment and water quality [2]. Freshwater leeches *Whitmania pigra* Whitman (*W. pigra*) are members of the Haemopidae family, Hirudinea class and Annelida phylum, widely found in East Asia. But due to pesticides, pollutants, and the disorderly fishing exploitation, the population of wild leeches has declined by more than 60% in the past decades. Knowledge on leech cocoon production and embryonic development may help the recovery of wild leech populations and be of great value to the research of annelids.

Glossiphoniid leeches of the genus Helobdella have been selected as a model organism representing Annelida in developmental biology studies since the 1970s. In 2001, with a clearer observation of the developmental process, Huang and Weisblat [8] standardized the naming of blast cells and summarized the developmental process into 11 stages. In situ hybridization analysis, which can display the temporal and spatial expression of genes, has been used to show the unique expression patterns of 13 *wnt* genes on the germinal plate in *Helobdella robusta* [9]. However, this approach relies on probe synthesis and therefore only a limited number of genes have been characterized. The whole-scale dynamic transcriptome changes during leech embryonic development are not yet available.

Adult leech (such as *Hirudo medicinalis*) has been used as a research model on neurobiology for its regeneration capability in the central nervous system, widely used to solve electrophysiological characteristics, behavior, treatment and development. The involvement of microglial cells in the neuronal regeneration was first shown in leeches [10]. Compared with leech *Helobdella robusta*, *W. pigra*’s embryos are more resistant to stress, relatively big and easily to obtain. *W. pigra* leeches are widely distributed in East Asia, while *Helobdella* are only distributed in America. A *W. pigra* weighing 20 g can produce 5.9 cocoons in average and each cocoon can hatch 37.3 leech seedlings [11]. The embryos grow by the nutrition supply of yolk and cocoon fluid, and develop into mature larvae in about one month. *W. pigra* also has experimentally accessible nervous system with ordered structure and a relatively small number of neurons. Its central nervous system consists of 34 ganglia, including 21 ganglia lined up in the middle of the whole body, and 6 head ganglia plus 7 tail ganglia at both ends of the body. Each ganglion has less than 200 pairs of neurons [12]. Identifiable by electrical characteristics and function, each neuron plays a specific and unique role [12]. The differentiation of these nerve cells started at stage 6a in leech embryos [13], but the genes involved in the leech neural differentiation are not clear. Thus, elucidating the molecular mechanism of embryonic development in *W. pigra* could potentiate *W. pigra* as a model for developmental biology.

To facilitate our understanding of the molecular mechanism underlying developmental events in *W. pigra*, we integrated genomic data of *W. pigra* adult and transcriptomic data of eight representative developmental phases of *W. pigra* embryos (from blastocyst to maturity). Utilizing a comprehensive transcriptome dataset, we screened the key transcripts and pathways in the developmental process and verified sequencing data by cloning and qPCR technology. Our results provide a better understanding of *W. pigra* development at the molecular level and lay a foundation for further functional research.

## Materials and methods

### Sample collection and observation

Adult *W. pigra* leeches were collected from a leech breeding base in Weishan Lake, Shandong Province. In order to obtain fresh cocoons containing early-stage embryos, pregnant leeches were reared in an artificial ceramic breeding tube, which comprises a first shell, a second shell and a joint part (Supplementary Figure S1). The first shell and the second shell cooperate with each other through the joint part to form at least one accommodation cavity for the leech to breed. The breeding tube was covered with wet towel with regular water spraying to keep humidity at about 60%. Cocoons were collected from the breeding tubes at regular intervals. For embryo collection, a small opening was cut at the top of a clean cocoon, and the embryos were pipetted out together with cocoon fluid for observation and sample collection. Embryos were observed under a stereomicroscope (MZ75 or M165FC, Leica), and images were taken with digital cameras.

### Genome sequencing and RNA-seq

Genomic DNA was extracted from muscle tissue dissected from the body of a single adult *W. pigra* leech using a tissue genomic DNA Extraction Kit (TIANGEN, China). Samples of embryos at defined developmental phases were frozen with liquid nitrogen and RNAs were extract using RNAprep pure Tissue Kit (TIANGEN, China) in accordance with the standard protocol. The quality and quantity of DNA and RNA were tested by Nanodrop 2000 spectrophotometer (Thermo, USA) and 1% agarose gel electrophoresis. The Reverse transcription system Kit (Promega, USA) was used to synthesize the first strand of cDNA.

For genome sequencing, 260 bp paired-end shotgun libraries were prepared for sequencing on an Illumina NovaSeq 4000 platform to generate an initial survey based on a 19-*K-mer* distribution. The software Jellyfish (v2.1.4) [14] was used for counting k-mers, and the software GenomeScope (v1.0) [15] was used for estimating the genome size. Through whole-genome sequencing, a DNA library was constructed using the standard protocol of PacBio. RNA-seq libraries were generated using Vazyme kit and sequenced using NovaSeq 6000 platform (Illumina, USA). Sequencing data have been deposited in the NCBI Sequence Read Archive.

### Genome assembly, annotation and RNA-seq data analysis

After filtering the low-quality and short clips of the raw fastq data from PacBio sequencing, the filtered data were corrected using Canu [16] and assembled with WTDBG (https://github.com/ruanjue/wtdbg). Error corrections were performed on the assembly result based on the Illumina sequencing data using Pilon [17]. BUSCO v5.5.2 [18] was used to evaluate the integrity of leech genome through sequence similarity (identity > 70%) alignment. Founded on an integrated strategy including ab initio, homology-based and transcriptome-assisted annotation, gene structure of the *W. pigra* genome was predicted. Specifically, Augustus (v3.1.0) [19] and SNAP (2006–07-28) [20] were used for ab initio prediction; GeMoMa (v1.7) [21] was used for gene prediction, based on six homologous species (*Amynthas cortices* [22], *Capitella teleta* [23], *Hirudo medicinalis* [24], *Helobdella robusta* [23], *Lottia gigantea* [23], *Whitmania pigra Ref* [25]); Three transcriptome [PRJNA931365, PRJNA903406 and PRJNA777652 (this study)] were used for transcriptome-assisted gene prediction with GeneMarkS-T (v5.1) [26] and PASA (v2.4.1) [27]. Results were integrated with EVM [28] software. RepeatMasker [29] and GeneWise [30] software were used to predict repetitive sequence and pseudogenes, respectively. Based on Rfam [31] database, rRNA were identified using Blastn for genome-wide alignment, microRNA were predicted using Infenal (v1.1) [32] against Rfam database (v 14.5) [31], and tRNA were predicted by tRNAscan-SE [33]. Gene annotation was performed through blasting against Gene Ontology (GO; http://www.geneontology.org/) [34], The Clusters of Orthologous Groups (COGs; http://www.ncbi.nlm.nih.gov/COG), Kyoto Encyclopedia of Genes and Genomes (KEGG; http://www.genome.jp/kegg) [35], Non-Redundant Protein Sequence Database (NR; ftp://ftp.ncbi.nih.gov/ blast/db/) [36, 37], Swiss-Prot (https://www.uniprot.org/) [38], EuKaryotic Orthologous Groups (KOG; http://www.ncbi.nlm.nih.gov/KOG/) and Pfam (http://pfam.xfam.org/) [39] databases. Analysis of orthologous clusters was performed on OrthoVenn2 (https://orthovenn2.bioinfotoolkits.net/gallery) [40].

Clean data of RNA-Seq was obtained by filtering off adaptor sequences, low-quality reads, duplicated sequences and poly-N from raw reads and then aligned with our reference genome of *W. pigra* using HISAT2 [41]. The aligned reads were assembled and evaluated using StringTie [42] software package. On the basis of the criteria of fold change > 2 and false discovery rate (FDR) < 0.05, differentially expressed genes were chosen using DEGseq [43]. Function information of genes were annotated in GO, COG, KEGG, nr, SwissProt, KOG, eggNOG and Pfam databases.

### Verification through cloning and real-time quantitative PCR

The target fragments to be verified were obtained by PCR. After ligating the target fragments with vectors and then transforming to *E. coli*, positive colonies were picked for plasmid preparation followed by sequencing using Sanger’s sequencing techniques. Quantitative PCR was performed using TB Green kit (Takara, Japan) on CFX96 Real-Time System (BIO-RAD, the U.S.A). The cycling parameters were set up as follows: step 1: 95 °C for 30 s; step 2: 95 °C for 5 s, 60 °C for 30 s and 72 °C for 30 s, performed with 40 cycles; step 3: (melt curve) 65–95 °C, increase by 0.5 °C every 5 s. The PCR reaction system was 25 μL: 12.5 μL of 2 × TB Green mix, 2 μL of the forward and reverse primers (2.5 μM), 2 μL of DNA template, made up the volume to 25 μl with sterilized ultrapure water. The specific primers used were synthesized by SANGON biotech company. The *α-tubulin* gene was chosen as the endogenous control for normalizing the relative expression content of genes using 2^−ΔΔCT^ method [44]. All primer sequences were listed in Supplementary Table S1 and S2.

## Results

### Embryonic development and phase definition of *W. pigra*

**Fig. 1:**
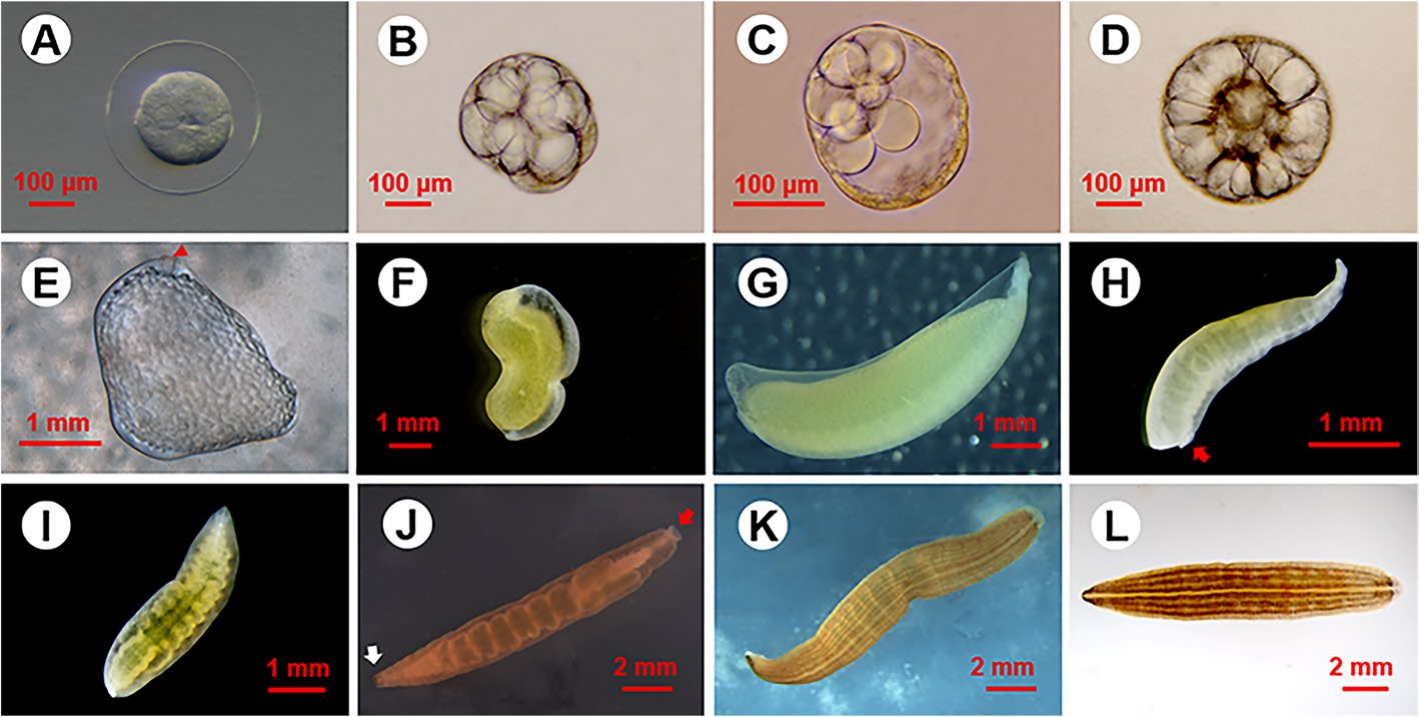
The process of embryonic development of *W. pigra*. **A-C** Cleavage stages: embryo undergoes spiral division along with the teloplasm unequal distribution; **D** Blastocyst stage: SYC wrapped by blastoderm; **E–F** Gastrula stage: the embryo has a strong deformation movement, the shape of the blastopore (red triangle) changes; D-V and A-P axes formed after gastrulation. **G-K** Organogenesis and refinement. The development events in this stage include the differentiation and refinement of crop ceca and intestine, caudal sucker (red arrow, H, J), and eye spots (white arrow, J); **L** Juvenile. yolk depletion in embryos, the color of the five longitudinal body surface lines was darker. Larvae leave the cocoon and begin to feed for the first time. SYC, syncytial yolk cell; D-V, dorsal–ventral; A-P, anterior–posterior

We observed the whole embryonic development process of *W. pigra* from egg-laying to complete larvae. The embryo first underwent the cleavage stage (A to C in Fig. 1) with two helical divisions for two cell cycles. As the teloplasm (yolk-deficient domains of cytoplasm) became unevenly divided, the axis of spiral division gradually leaned towards the animal pole. After the cocoon formation at about four days later, the embryo reached the blastocyst stage (D in Fig. 1). The blastopore could be observed, and it will develop into the future mouth. After multiple mitoses, blastoderm became thinned gradually and the syncytial yolk cell (SYC) was tightly surrounded in the middle. The embryo underwent drastic morphogenetic movement, with the blastopore opening and closing to exchange materials with cocoon fluid (E in Fig. 1). The epiboly of germinal band marks gastrulation, after which coelomic cavities arose in a front-to-back progression and the embryo developed a clear dorsal–ventral axis and an anterior–posterior axis (F in Fig. 1). Figure 1H to K is the stages of organogenesis and refinement of leech. The boundary of crop ceca and intestine became clearly distinguishable, as the result of refinement of structures. During these phases, the posterior sucker differentiated into cup shape and extended to the body width. Eleven pairs of eye spots became gradually pigmented. The nervous system developed, with the ganglions at the midline of the back clearly visible (Fig. 1K). Figure 1L shows a leech larva that resembles the adults, with well-defined segments, muscle and organs. Compared to phase, the most obvious difference of L was the deepening of yellowish longitudinal lines with black spots on the back. At this phase, the yolk in the embryo was exhausted and leech larvae can crawl out of cocoon for their first feeding.

### Genome sequencing and assembly

Based on 19-*k*-mer analysis, we estimated that the *W. pigra* genome was to be 219.40 Mb in size with a medium level of repetition (33.27%) and heterozygosity (0.61%). We used a total of 80.0 × coverage of single-molecule sequences of the PacBio Sequel platform for assembly, and generated 350.0 × Illumina data for sequencing error corrections and gap filling. The size of the final assembly was 178.87 Mb, with 483 contigs and a contig N50 of ~ 2.00 Mb (Supplementary Table S3). We then used BUSCO analysis to estimate the completeness of genome assembly, and obtained a completeness score of 95.7% (Supplementary Table S4). From the genome assembly, we predicted 25,169 high-quality protein-coding genes, and 456 pseudogenes due to frameshifts or premature stop codons. The average length of protein-coding genes was 4194.64 bp, and the mean sequence length of exons was 1738.44 bp. Furthermore, noncoding RNA genes were predicted, yielding 738 tRNA, 51 rRNA and 164 miRNA (Supplementary Table S5). We annotated a total of 69.07 Mb of repetitive elements in the *W. pigra* genome. Results showed that 84.44% of the genes can be annotated into nr, nt, GO, KEGG, pfam, TrEMBL, KOG, COG databases.

**Fig. 2:**
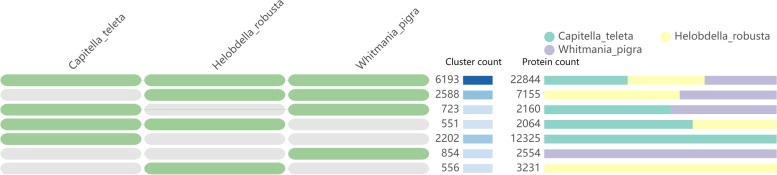
Comparison of genome-wide orthologous gene clusters in three related organisms. The number of clusters was sorted by overlap. The number and distribution of proteins in clusters are shown in the figure. Green, yellow and purple represent *Capitella teleta*, *Helobdella robusta* and *W. pigra* respectively

We used OrthoVenn2 to compare the translation coding sequences of *Capitella teleta*, *Helobdella robusta* and *W. pigra* for homologous genes. A total of 13,667 clusters were analysed, of which 8895 orthologous clusters (at least contains two species) and 4772 single-copy gene clusters (Fig. 2, Supplementary Table S6). The annotation shows that cluster2, cluster3 and cluster6 with the most genes are related to intracellular signal transduction, apoptosis and nucleosome assembly (Supplementary Table S7).

### RNA sequencing, mapping and new transcripts exploiting

We collected specimens from eight representative phases of the *W. pigra* embryonic development, covering the starting point of cell differentiation to individual maturation, corresponding to D-L (except for K) in Fig. 1 (see Table 1) for RNA sequencing. After quality control, we obtained a total of 239.64 Gb clean data from 24 samples, with each sample reaching 6.14 Gb of data. The Q30 base was no less than 91.18% and the mean GC content was 44.37% (Supplementary Table S8). Results from StringTie showed that the average comparison efficiency between RNA-seq reads and our reference genome was 81.15%. And 84.9% reads were distributed in exon regions, indicating that most of reads were located on mature mRNAs. Totally, 21,482 genes were identified in our transcriptome, 19,765 were present as known or predicted transcripts in major transcriptome databases, such as Pfam, eggNOG, nr, COG, GO, KEGG. We discovered 48 new transcripts that were not annotated in the genome assembly.

**Table 1:** Selected eight representative phases for sequencing (*N* = 3)

Phase	Representative developmental events
D	Blastocyst stage
E	Severe deformation and epiboly
F	Coelomic cavities formation
G	Distinct dorsoventral pattern, anterior–posterior axis
H	The organogenesis and refinement of crops, intestine, eyes and posterior sucker
I	
J	
L	Body surface lines

### Differential expression analysis of *W. pigra* embryonic transcriptome

**Fig. 3:**
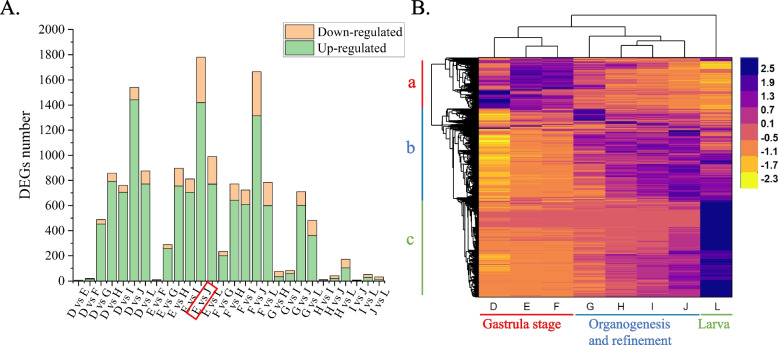
**A** Barplot displaying the number of differentially expressed genes in paired comparison. **B** Heat maps of DEGs show that gene expression patterns can be divided into three branches both vertical and horizontal

To gain insights into the dynamic gene expression profiles in different phases of embryonic development of *W. pigra*, we performed pairwise comparison with fold change ≥ 2 and FDR ≤ 0.05 as cutoff values for differentially expressed genes (DEGs). In total, 15,161 DEGs were identified in the comparison of 28 groups (Table 1), of which 12,671 were up-regulated and 2490 down-regulated. After removing duplications among groups, 3114 DEGs were obtained, which accounted for approximately 13% of the leech transcriptome. The number of DEGs was the smallest in E vs F phase (early gastrula stage), J phase contained the largest number of DEGs, followed by L phase (Fig. 3A). This suggested that the differentiation begins at the blastocyst stage and reaches the peak at the time of organ differentiation. The heatmap of DEGs (Fig. 3B) shows obvious phase-specific expression patterns both vertically and horizontally. D-F phases represent gastrula stage, cluster (a) genes were uniquely highly expressed in D-F phases; genes in G-J phases share similar expression patterns, participating in organogenesis and refinement; L phase represents developed larva, genes in cluster (c) were only highly expressed in L phase.

**Fig. 4:**
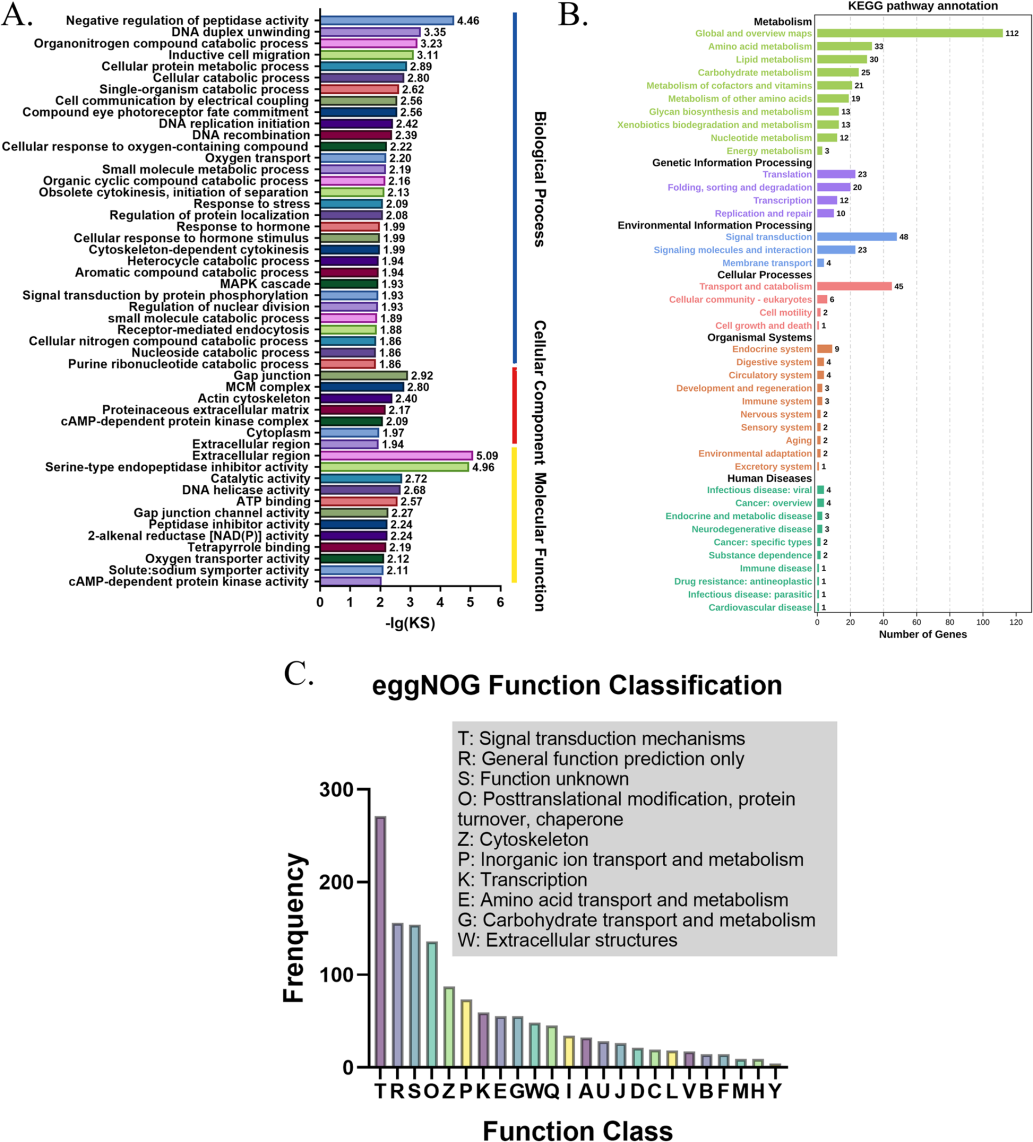
Differential expression analysis of E vs J phase. **A** Functional annotation in GO databases. The results show that DEGs were annotated in biological process, cellular component and molecular function. **B** Analysis of expression in KEGG database. **C** Functional annotation in eggNOG databases. (*Q: Secondary metabolites biosynthesis, transport and catabolism; I: Lipid transport and metabolism; A: RNA processing and modification. U: Intracellular trafficking, secretion, and vesicular transport. J: Translation, ribosomal structure and biogenesis. D: Cell cycle control, cell division, chromosome partitioning; C: Energy production and conversion. L: Replication, recombination and repair; V: Defense mechanisms; B: Chromatin structure and dynamics; F: Nucleotide transport and metabolism; M: Cell wall/membrane/envelope biogenesis; H: Coenzyme transport and metabolism; Y: Nuclear structure*)

We performed functional annotation of DEGs and found that E vs J group contains most of the DEGs. According to the annotation results of GO database, the top 5 terms in biological process were negative regulation of peptidase activity, DNA duplex unwinding, organonitrogen compound catabolic process, inductive cell migration and cellular protein metabolic process. This showed that the energy required for embryonic development mainly comes from the decomposition of organic nitrogen. DNA unwinding represents the rapid progress of cell division. The process of cell communication and migration promotes tissue and organ differentiation. Under cellular component category, the first three terms were the gap junction, MCM complex and actin cytoskeleton. Gap junction, a special membrane structure between cells, are critically important in many biological activities, including development and differentiation [45]. MCM2-7 complex is essential to DNA duplication, assembled on chromatin in G1 phase of mitosis and separated during S phase [46]. Molecular function was mainly related to extracellular region, serine-type endopeptidase inhibitor activity and catalytic activity. EggNOG enrichment analyses revealed that, the function of J phase is mainly related to signal transduction mechanisms, posttranslational modification, protein turnover, chaperones and cytoskeleton. KEGG annotation showed “global and overview maps”, “translation”, “signal transduction”, transport and catabolism” and “endocrine system” lead the rankings. These results indicated that the development of *W. pigra* is closely associated with metabolism, cell structure, cell proliferation and differentiation (Fig. 4).

To identify species specificity, we searched all of the assembled unigenes against the NR database ((Supplementary Figure S2). This showed that Glossiphoniid leech *Helobdella robusta* shares the most of homologous protein with *W. pigra* (56.68%), followed by Annelida *Capitella teleta* (11.35%) and *Ceratitis capitata* (4.15%).

### Genes involved in morphogenesis during *W. pigra* embryonic development

**Fig. 5:**
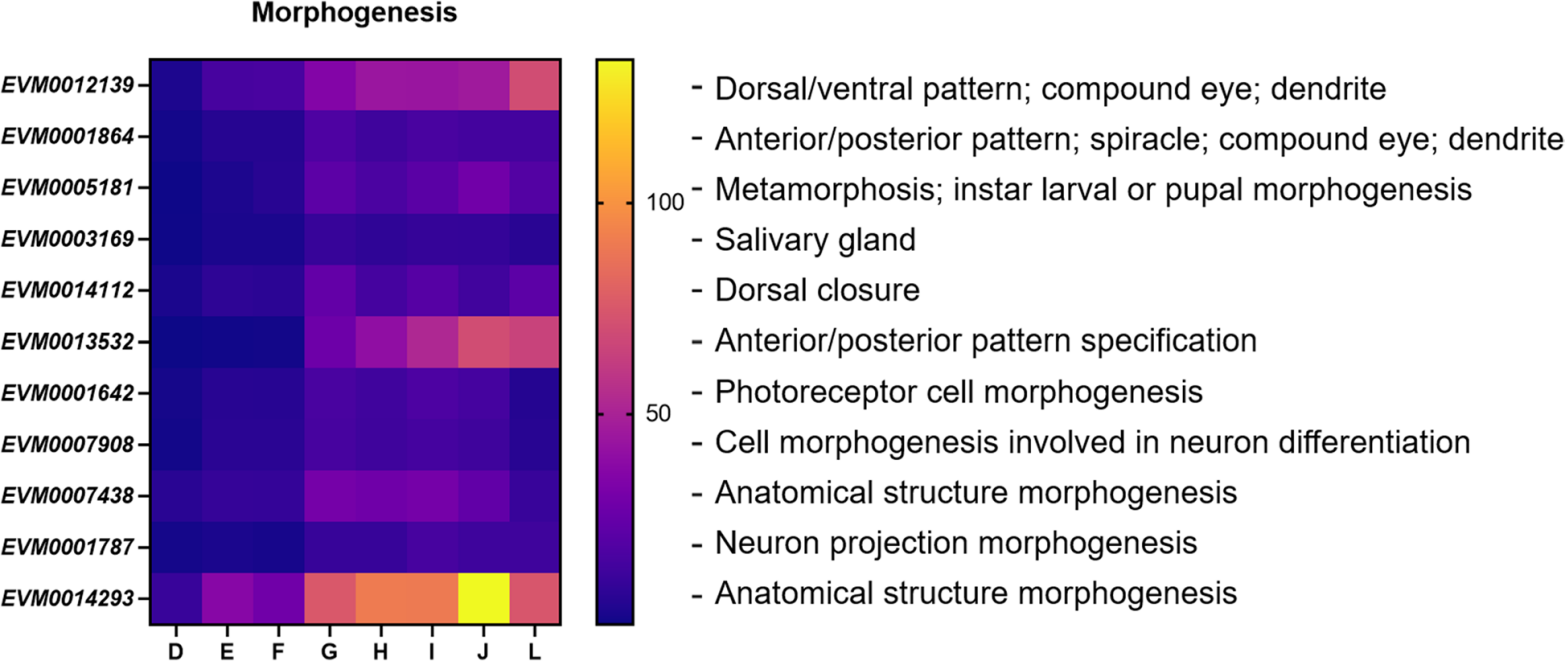
DEGs related to morphogenesis during embryonic development of *W. pigra* leech (D-L phases)

The above pairwise comparison highlights the dynamic gene expression pattern in *W. pigra* embryonic development. From the perspective of the GO terms related to “morphogenesis” at the eight developmental phases of *W. pigra*, we identified 57 significantly DEGs (Supplemental Table S9), and selected 14 representative genes for further analysis (Fig. 5). These genes were expressed at very low level across early developmental periods while highly expressed in G-J phases. From the annotation results of the GO database, gene functions include dorsal/ventral pattern formation, eye development, dendrite morphogenesis, dorsal closure, cell morphogenesis involved in neuron differentiation and so on.

The formation of dorsal–ventral axis and antero-posterior axis is the most obvious morphological feature during *W. pigra* embryonic development. It has been shown that *EVM0012139*, encoding Fascin (80.4% similar with *Helobdella robusta*), is ubiquitous in early embryonic development, contributing to dorsal/ventral pattern formation and the growth of muscle, dendrite and other cells. It was highly expressed in phase G and peaked in phase L. Similarly, gene *EVM0013532* and *EVM0001864* was annotated to participate in the formation of anterior/posterior pattern. *EVM0001864* has low identity with characterized protein, the similarity with evolutionarily conserved C2H2-type zinc finger protein is about 30% and it was annotated as transcriptional activator in SwissProt database. Therefore, we suggested it plays a role in the development and differentiation of tissues/organs as a transcription factor. It maintained uniform and stable expression during the eight phases of development. Matrix metalloproteinase translated by *EVM0005181* has proteolytic activity and an effect on signal factors, so its high expression is closely related to embryonic development [47]. It is important to note that the presence of the salivary gland is of significant value to the medicinal use of leeches. *EVM0003169* was annotated as a matrix metalloproteinase 1 isoform X3 promoting the morphogenesis of the salivary gland. *EVM0014112* was highly expressed in G phase, mainly involved in the development of dorsal closure through protein interactions mediated by the FERM domain. *EVM0001642* had to do with the differentiation of photoreceptor cells, expressed in the G-J phases. This indicates that the photosensitive ability of *W. pigra* develops before maturity. The dynamic expression profile of *EVM0001787* indicated that the cell morphogenesis involved in neuron differentiation mainly occurs during G to L phases. In addition, gene *EVM0007438* and *EVM0014293* were related to immunoglobulin domains. These two genes were both highly expressed in the late period of leech, especially *EVM0014293*, with the highest FPKM value reached 133.90, indicating their important roles in immune system.

### Signal pathways in *W. pigra* embryonic development

**Fig. 6:**
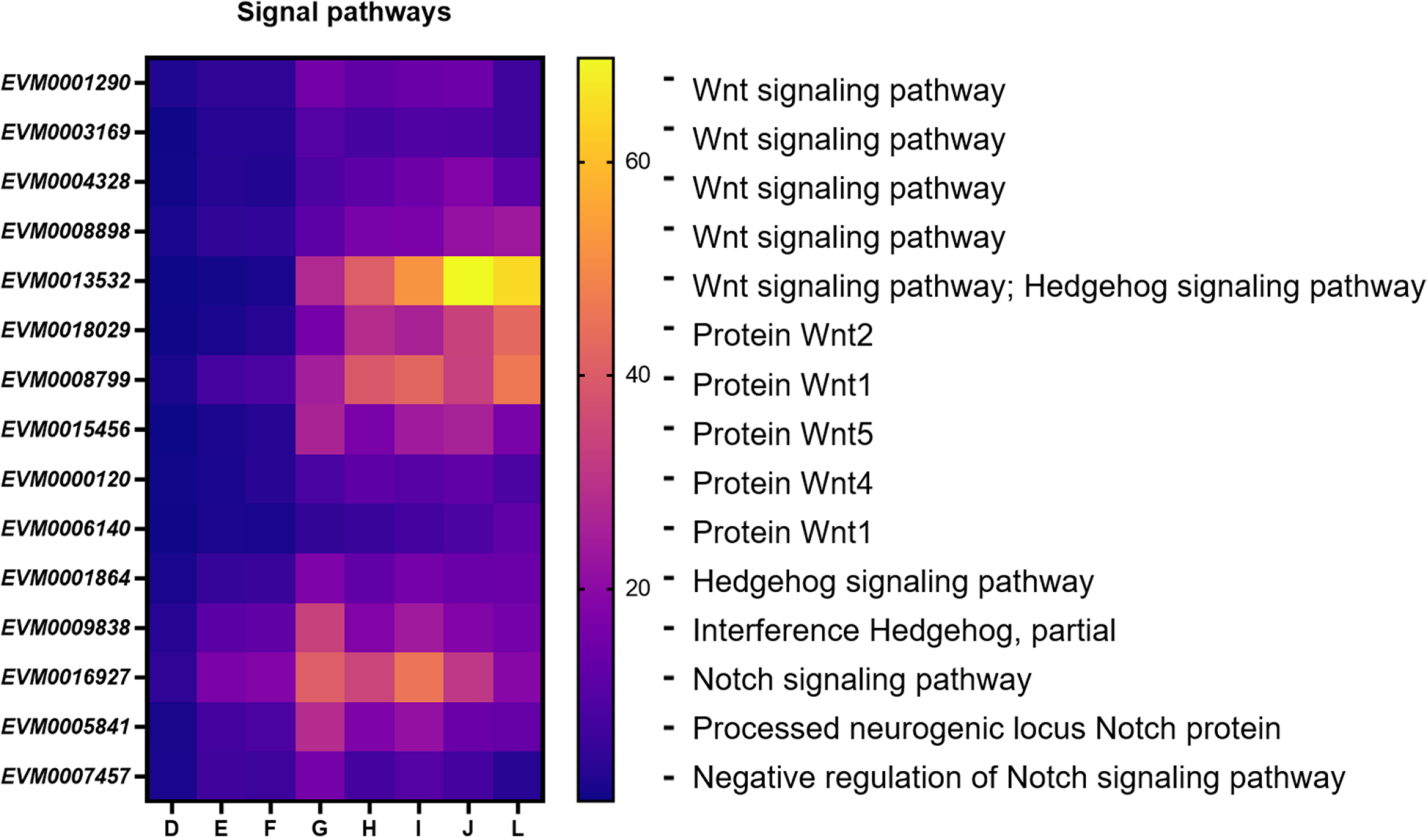
DEGs related to signal pathways during embryonic development of leech (D-L phases)

Among all the DEGs, 49 genes were related to Wnt pathway, Hedgehog pathway and Notch pathway (Supplemental Table S10). We selected fifteen genes for further detailed analysis. This included 10 *wnt* genes, 3 *notch* genes and 2 *hedgehog* genes (Fig. 6). Instead of having a high expression in specific periods like morphogenesis genes, genes related to signal transduction were highly expressed throughout the developmental process. This suggested that these genes undertake different developmental tasks throughout the whole developmental process.

*Wnt* and *hedgehog* genes were all expressed at a low level in E–F phases, followed by a significant growth in phase G, indicating Wnt cooperated with Hedgehog participated in blastoderm differentiation and organogenesis. Here, we explicitly annotate six Wnt family members, including Wnt1, Wnt2, Wnt4, Wnt5, Wnt16 and Wnt16B (Wnt16 and Wnt16B are shown in Supplemental Table S10). From the perspective of gene expression, Wnt1, Wnt2 and Wnt16 were widely involved in the development of G-J phases, and still play a great role after leech maturity. Wnt4 and Wnt5 mainly regulate embryonic development, and the expression level decreased after maturation.

**Fig. 7:**
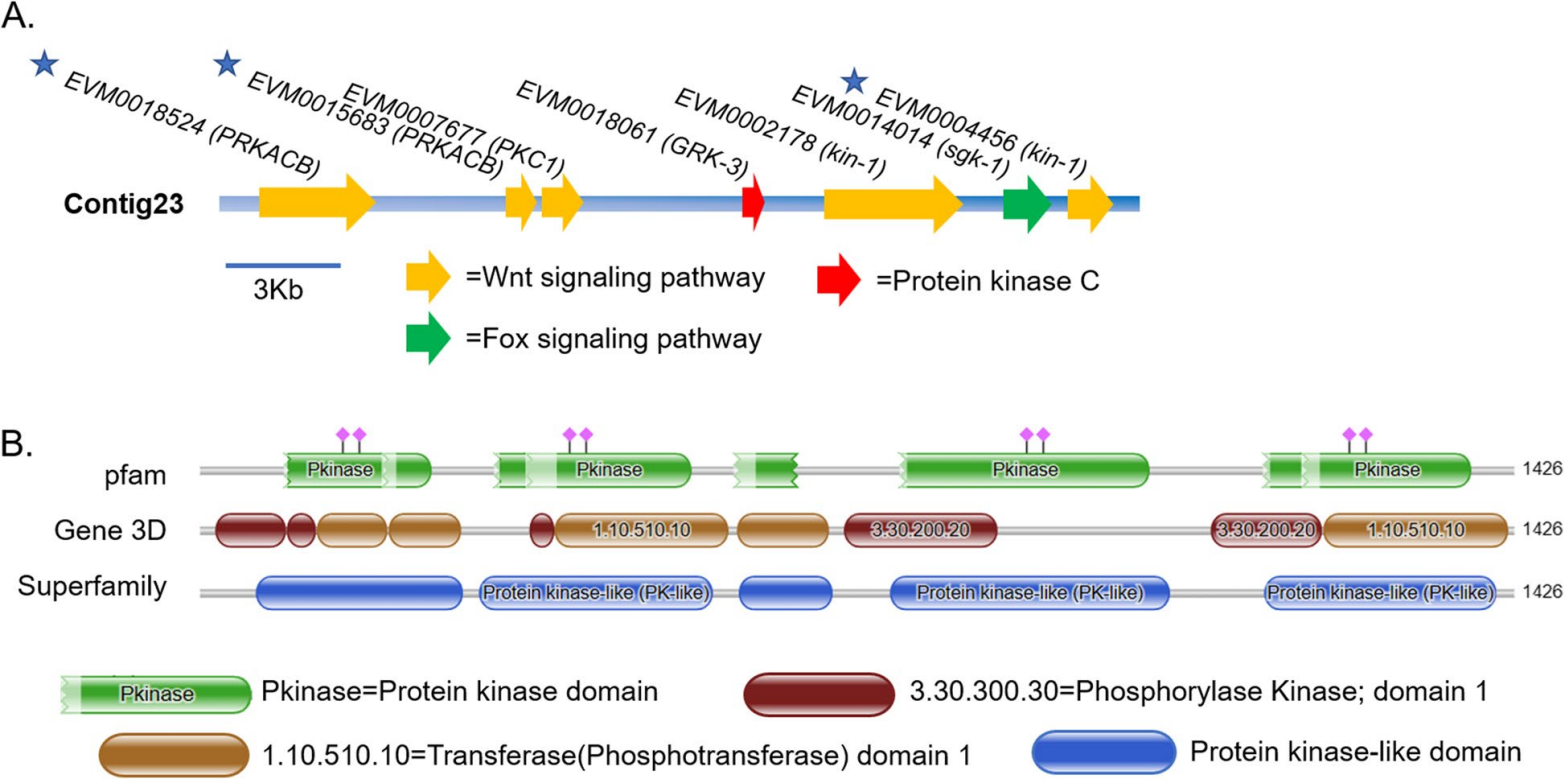
A gene cluster related to Wnt signaling pathway in contig23. **A** Seven genes are closely distributed in contig23. Asterisks indicate three genes involved in synaptic pathways. **B** The predicted arrangement of the protein domains

In addition, a 21.7-Kb-long sequence was found related to signal pathway (Fig. 7A), including seven genes (*EVM0018524*, *EVM0015683*, *EVM0007677*, *EVM0018061*, *EVM0002178*, *EVM0014014* and *EVM0004456*). The gene interval between these genes was no more than 3979 bp. Five kinase domains and five transferase domains were predicted (Fig. 7B), suggesting that this sequence may have a role in a number of cellular functions. Therefore, these genes can be considered as a gene cluster which potentially perform the same function. Moreover, genes *EVM0018524*, *EVM0015683*, and *EVM0004456* are associated with five distinct synaptic pathways, which include glutamatergic, cholinergic, serotonergic, GABAergic, and dopaminergic pathways.

Genes *EVM0001864* and *EVM0009838* are related to Hedgehog signaling. *EVM0001864* has conserved C2H2-type double zinc finger domain, playing a role in transmitting Hedgehog signals and morphogenesis. *EVM0009838* was highly expressed in G phase. Its encoding protein includes four kind of Immunoglobulin domain (IgV, IgC1, IgC2, and IgI) and fibronectin type III domain.

In the middle stage, three notch genes (*EVM0016927*, *EVM0005841*, and *EVM0007457*) were expressed more highly than in the early and late stages. As a component of the pathway responsible for the formation of the dorso-ventral axis (Ko04320), *EVM0016927* plays a key role in the formation of pattern. *EVM0005841* encodes the Calcium-binding EGF domain, which usually imparts specific functions to proteins in the coagulation cascade. Here was annotated as a processed neurogenic locus Notch protein. *EVM0007457* was annotated to negatively regulate the Notch signaling pathway, with a similar change trend of gene expression horizontal as *EVM0005841*. It suggested the activation and inhibition of the Notch pathway are regulated simultaneously to regulate the normal growth of embryos.

### Neural development in *W. pigra*

**Fig. 8:**
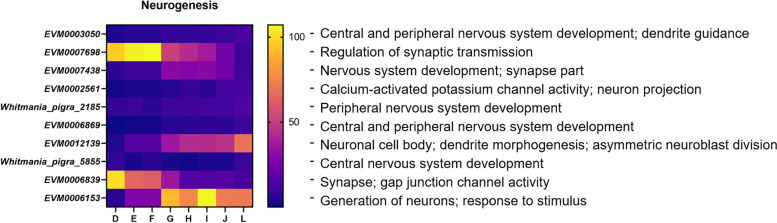
DEGs related to neurodevelopment during D-L phase

Our annotation results of all DEGs showed that 77 genes were assigned to “neurogenesis”, taking part in the development of the central nervous system, peripheral nervous system or neural structure of leeches (Supplemental Table S11). Here, 10 representative genes and their expression data throughout eight developmental phases are listed in Fig. 8.

*EVM0003050* was expressed at its highest level in the L phase, its coding protein Homeobox influences the transcription process to play a role in central and peripheral nervous system development. *EVM0002561* encodes calcium-activated potassium channel protein, determining neuron projection morphogenesis. Similar functions include calcium ion binding (*EVM0000129*), neurotransmitter-gated ion-channel ligand binding domain (*EVM0005725*), calcium-activated potassium channel (*EVM0016702*), and so on. Tyrosine phosphatase genes usually participate in the post-translational modification for neurogenesis and motor neuron axon guidance. *EVM0007698* encodes protein-tyrosine phosphatase, it was highly expressed in D-F phase, suggesting its role in the early-stage regulation of synaptic transmission. *EVM0006869* serves as a neurogenic locus protein with a low expression level. It contributes to the development of the central nervous system and the peripheral nervous system. Significantly, eight genes encode Innexin (such as *EVM0006839*), functioning in gap junction and synapse. *EVM0007438* is related to the Immunoglobulin domain, its encoding protein fasciclin-2 is a neural cell adhesion molecule, widely involved in the development of the nervous system. *Whitmania_pigra_newGene_2185* is related to muscleblind-like protein, contributing to the development of the peripheral nervous system. Protein Singed and Glass were coded by *EVM0012139* and *Whitmania_pigra_newGene_5855* respectively, playing an important role in neurogenesis and central nervous system development.

**Fig. 9:**
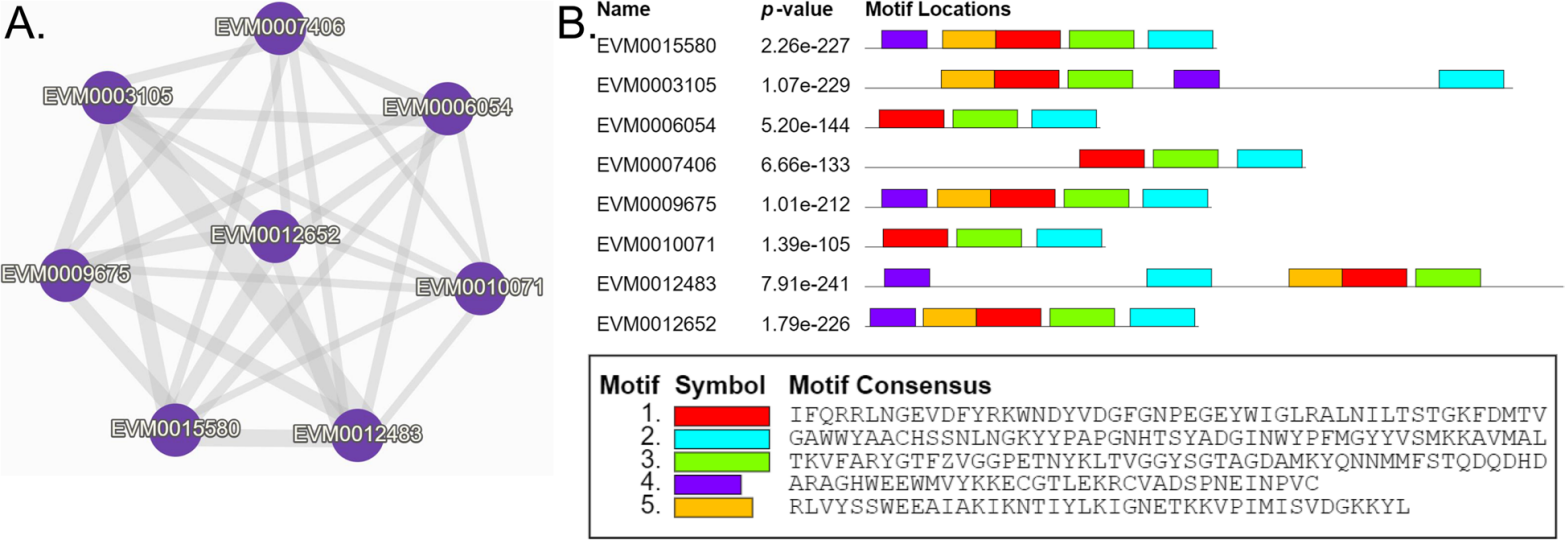
A gene cluster containing 8 genes regulated neurogenesis during embryonic development of *W. pigra*. **A** shows the relationship of 8 genes, each edge represents the similarity between two protein sequences. **B** presents conserved domain analysis of genes using MEME

**Fig. 10:**
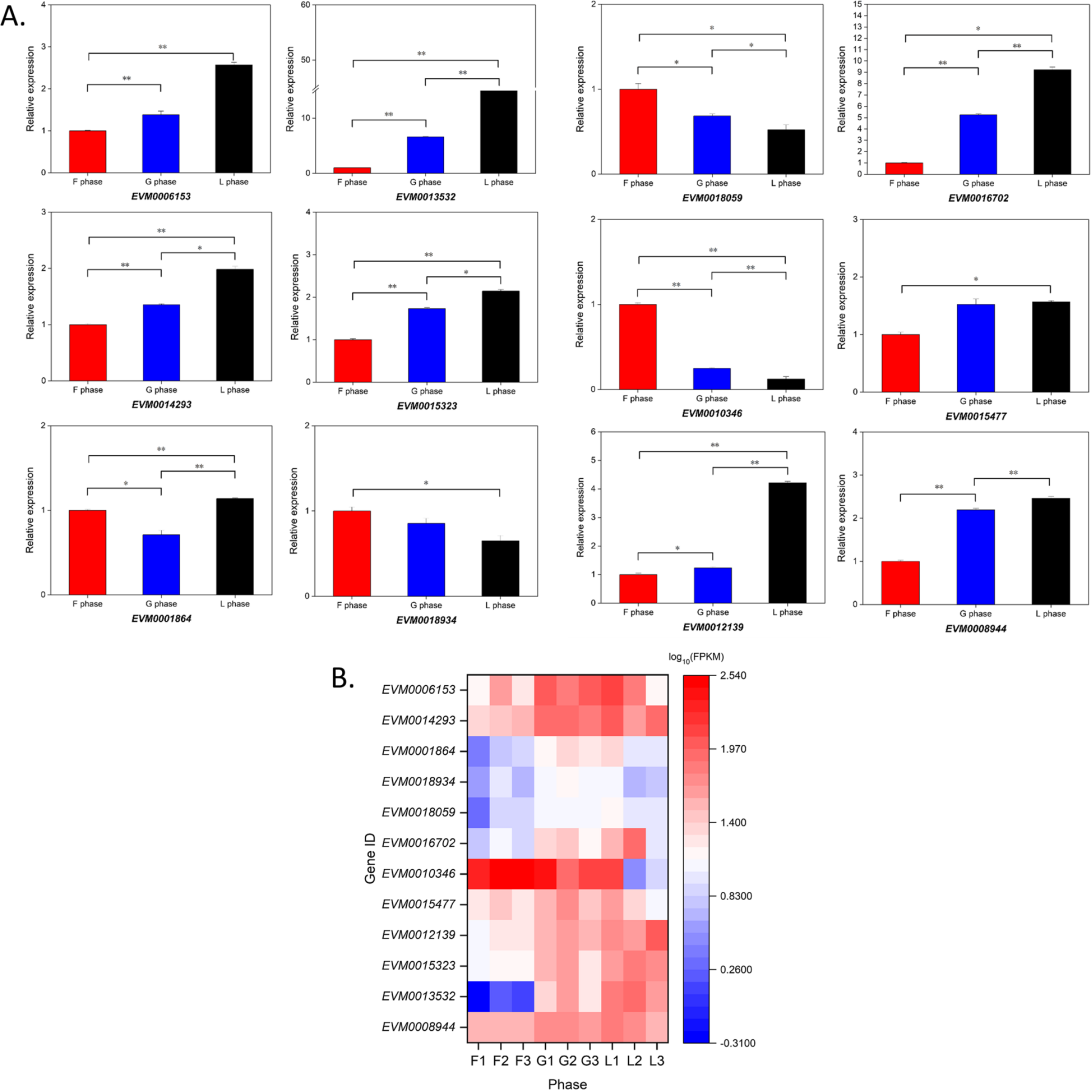
**A** Relative expression of 12 genes related to morphogenesis and neurogenesis using qPCR analysis. Red, blue and black color indicates the expression of genes in F, G and L phase (*N* = 3). **B** Heatmap of 12 genes obtained by RNA-seq

Eight genes are found in the genome to be divided into a cluster involved in the regulation of neurogenesis (Fig. 9A). The relationship between the eight genes was shown in Fig. 10. *EVM0003105* had the highest similarity with *EVM0012483*. MEME software was used to predict genes in this cluster and the distribution of five conserved motifs were listed (Fig. 9B). A similar arrangement of motifs was found in *EVM0015580*, *EVM0009675* and *EVM0012652*.

### Gene verification by gene cloning and qPCR

To validate our RNA sequence data, we successfully cloned twenty-nine genes into plasmids and sequenced them, including 16 genes related to signal pathways, 10 genes related to signal transduction mechanisms, and three genes related to dorsal–ventral pattern, myostatin and TGF-beta propeptide respectively. In addition, we verified 12 genes (related to morphogenesis and neurogenesis) by qPCR experiment. The results showed that the expression pattern determined by qPCR was consistent with the results of RNA sequencing (Fig. 10), confirming the reliability of the transcriptome analyses.

## Discussion

*W. pigra* is a classical traditional Chinese medicine component for promoting blood circulation and removing blood clots. With the rapid increase of annual demand comes the need for refining the breeding process, from temperature and humidity adjustment, to water quality control, breeding site selection, and cocoon production monitoring, in order to maximize production. However, understanding of *W. pigra* embryo development is an important gap in leech farming. This is also important for leech breeding, yield improvement, and resistance enhancement. We observed the embryonic development process of *W. pigra* and divided the development process of *W. pigra* into eight phases according to the morphological characteristics. The criteria we used to divide phases can be compared with the standard 11 phases, despite that we paid more attention to the gross morphological changes rather than the precise cell lineages, which is more difficult for *W. pigra*. This criteria has been verified by Pearson's correlation coefficient (r) analysis [48] of RNA sequencing data. Results showed that r^2^ value was close to 1, suggesting that the expression similarity of biological duplicates is high enough for subsequent DEGs analysis.

The combination of *W. pigra* genome and transcriptome facilitates the analysis of genome neighborhood environment of genes with specific functions because metabolic pathways are often encoded by proximal genes (operons and/or gene clusters) [49]. We have compiled a set of procedures to screen out genes with a distance of less than 3000 bp and on the same KEGG pathway (Supplementary file 12). The cluster on contig23 mentioned above contains seven genes, *EVM0018524* and *EVM0015683* encode cAMP-dependent protein kinase catalytic subunit beta (PRKACB), active PRKACB regulated cell proliferation [50], differentiation [51], cell cycle, migration [52] and other processes. *EVM0007677* (PKC1) has been implicated to regulate peptide neurosecretion in *Caenorhabditis elegans*, however there is no experimental evidence of a developmental relevance. *EVM0018061* (GRK-3) encode G protein-coupled receptor kinase. Kin-1 (*EVM0002178*, *EVM0004456)* is essential for larval development. Polypeptides resulting from alternative splicing from kin-1 has been found to have substantial differences in embryonic and adult nematodes [53]. *EVM0014014 (SGK-1)* is the crucial kinase for the control of development, stress response, and longevity [54]. In a word, this cluster is essential for *W. pigra* embryonic development. While only one gene cluster related to signal pathways was shown in the current study, other gene clusters will be further explored.

Using a comprehensive transcriptome dataset, we obtained the dynamic transcriptome changes that occurred from blastocyst stage to maturity. We observed that genes related to dorsal/ventral and anterior/posterior formation are highly expressed in G-J phases, while genes related to salivary gland differentiation and photoreceptor differentiation are dynamically expressed in H phase. Generally speaking, a complex signal network regulates the process of organogenesis and the induction of morphological characteristics [55]. Studies have shown that Wnt signaling cascade with Notch, and Hedgehog signaling to regulate the balance of embryonic cell [56]. As speculated, most of *wnt*, *notch* and *hedgehog* are highly expressed in G-H phase of *W. pigra* embryos.

Many genes are involved in the regulation of more than one signal pathways, such as *EVM000120* and *EVM0013532*, indicating that these signal pathways play a coordinated role in embryonic development. Notably, our results suggest that *wnt* gene *EVM0003169* plays an important role in the regulation of salivary gland development. Studies have compared the transcriptome levels of the salivary glands of leech before and after blood sucking, and identified the top 20 KEGG pathway including cAMP, MAPK and PI3K-Akt signal pathway, except for the Wnt pathway [57]. Therefore, we speculate that the Wnt signal pathway is of great significance for the development of salivary glands but not responsible for the production of active substances in saliva.

During neural development of *W. pigra*, the most abundant genes were Homeobox genes (11 genes), and there are studies showing that Homeobox gene families exhibit a regional distribution near the anterior posterior body axis in leeches, and the main function is about morphogenesis [58, 59]. It is tempting to speculate, based on our results, that the Homeobox genes are involved in the development of the *W. pigra* nervous system. The detection of genes and substances can be achieved through electrochemical methods, such as Electrostatic self-assembly of MXene on ruthenium dioxide modified carbon cloth [60]. The phosphorylation process of proteins is the final step to transmit information between nerve cells. Previous literatures show that phosphorylases and kinases can also affect the growth and development of nerve cells [61, 62]. *EVM0007698* encoding tyrosine phosphatase protein is highly expressed in D-F phases, likely participating in the construction of nerve cells. Meanwhile, molecular studies on the development of the leech nervous system also found many substances containing the Ig domain [63], which further supports the necessity of this domain. Secondly, studies in mice have shown that Fascin protein is expressed in the sciatic nerve and the hippocampal nerve, and the actin binding domain of Fascin protein can induce axonal extension [64]. *EVM0007438* has similar functional annotation, mainly promotes the development of synaptic part of central nerve during G-I phases.

## Conclusions

This study presents a high-quality genome of *Whitmania pigra*, and reports a transcriptome covering the starting point of cell differentiation to individual maturation. We described the whole process of *W. pigra* embryonic development and divided the phases according to morphological characteristics. We annotated a total of 21,482 protein-coding genes from the *W. pigra* genome and transcriptome data, with 3114 genes differentially expressed during development. We mainly focused on genes related to morphogenesis, signal pathway and neural development, and analyzed 57, 49 and 77 DEGs in detail. We found a cluster including seven genes related to signal pathways in larval development. We also verified the reliability of the data by gene cloning and qPCR experiments. Our work provides a rich resource for further analysis of *W. pigra* development.

## Supplementary Information

**Additional file 1:** **Figure S1.** Ceramic breeding tube for leech.

**Additional file 2:** **Figure S2.** Species distribution analysis in NR database.

**Additional file 3:** **Table S1.** Primers used in real-time PCR.

**Additional file 4:** **Table S2.** Primers used for gene cloning.

**Additional file 5:** **Table S3.** Sequencing and assembly statistics of the draft genome of Whitmania pigra.

**Additional file 6:** **Table S4.** BUSCO assessment of the Whitmania pigra genome.

**Additional file 7:** **Table S5.** List of non coding RNA (miRNA, tRNA, rRNA, snRNA).

**Additional file 8:** **Table S6. **All cluster(one line is one cluster) analysed by OrthoVenn2.

**Additional file 9:** **Table S7.** Annotation of clusters performed by OrthoVenn2.

**Additional file 10:** **Table S8.** RNA-Seq results of eight phases.

**Additional file 11:** **Table S9.** 57 genes related to morphogenesis.

**Additional file 12:** **Table S10.** 49 genes related to signal pathways.

**Additional file 13:** **Table S11.** 77 genes related to neurogenesis.

**Additional file 14:** **Supplementary file 12.** A program that filter clusters in the same KEGG pathway.

## Data Availability

The high-throughput sequencing datasets presented in this study can be found in the National Center for Biotechnology Information (NCBI) SRA online repository. The accession number of genome is ASM2161333v1 (https://www.ncbi.nlm.nih.gov/bioproject/?term=ASM2161333v1). The accession number of transcriptome is PRJNA777652 (https://www.ncbi.nlm.nih.gov/bioproject/PRJNA777652). The accession numbers of the BioSample specimens are SAMN22870527-SAMN22870550. And the SRA accession numbers for the above 24 BioSample specimens are SRR16775025-SRR16775048, respectively.
