# Supplementary material for: Division of developmental phases of freshwater leech Whitmania pigra and key genes related to neurogenesis revealed by whole genome and transcriptome analysis
Source: BMC Genomics. 2023 Apr 17;24:203. doi: 10.1186/s12864-023-09286-5 (PMC10111769; doi:10.1186/s12864-023-09286-5)
Supplement: Supplementary file 1 — Additional file 1: Figure S1. Ceramic breeding tube for leech. [file 12864_2023_9286_MOESM1_ESM.pdf]

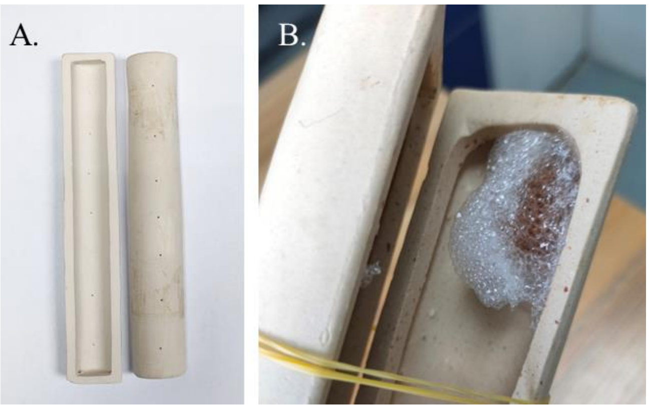


Figure S1. Ceramic breeding tube for leech. (A) The panorama of breeding tube, including a first shell, a second shell and a rubber band used to fix shells, there are spiracles at tube wall. (B) A leech produced a cocoon in the breeding tube for less than five hours, and the honeycomb-like structure on the surface of the cocoon was still not completely dry yet and the interior is pink.
