# Supplementary figures and images for: Division of developmental phases of freshwater leech Whitmania pigra and key genes related to neurogenesis revealed by whole genome and transcriptome analysis
Source: BMC Genomics. 2023 Apr 17;24:203. doi: 10.1186/s12864-023-09286-5 (PMC10111769; doi:10.1186/s12864-023-09286-5)

### NR Species distribution

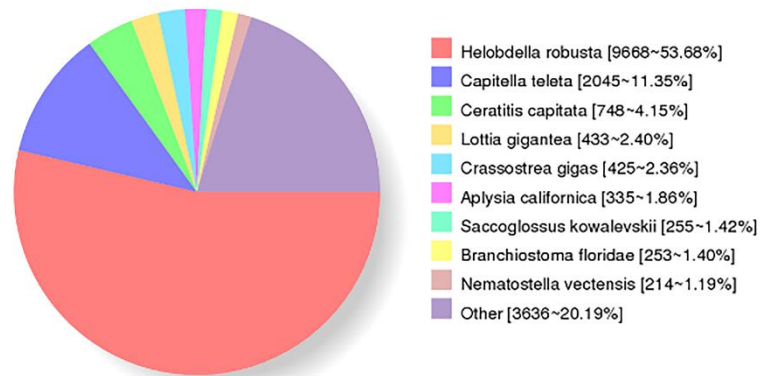

Figure S2. Species distribution analysis in NR database.

Supplement: Supplementary file 2 — Additional file 2: Figure S2. Species distribution analysis in NR database. [file 12864_2023_9286_MOESM2_ESM.pdf]
