# Supplementary material for: Division of developmental phases of freshwater leech Whitmania pigra and key genes related to neurogenesis revealed by whole genome and transcriptome analysis
Source: BMC Genomics. 2023 Apr 17;24:203. doi: 10.1186/s12864-023-09286-5 (PMC10111769; doi:10.1186/s12864-023-09286-5)
Supplement: Supplementary file 14 — Additional file 14: Supplementary file 12. A program that filter clusters in the same KEGG pathway. [file 12864_2023_9286_MOESM14_ESM.docx]

#encoding = utf-8

import sys

import os

#############################################

gene2contigFile = sys.argv[1]

cluster_dist = sys.argv[2]

cluster_dist = int(cluster_dist)

annotationFile = sys.argv[3]

outHandle = sys.argv[4]

print ("version: 20230208")

print ("Usage: python find_geneCluster.py gene2contigFile cluster_dist annotationFile outHandle")

print ("gene2contigFile is a four columns file separated by tab: geneid contigname gene_start_pos gene_end_pos.\n sample file:geneid_contigid_start_end.txt")

print ("cluster_dist is the maximum distance between genes in the sample cluster，for example 3000")

print ("annotationFile is a two collumns file separated by tab: geneid annotation.\n sample file: gene2annotation.txt")

print ("outhandle in the prefix of outfiles")

#################################################

D_annotation = {}

for line in open(annotationFile):

line = line.strip()

if line:

gene_id = line.split("\t")[0]

gene_info = line.split("\t")[1]

if gene_id not in D_annotation.keys():

D_annotation[gene_id] = gene_info

def extractCluster(L_genePos, cluster_dist):

#L_genePos

L_cluster = []

L1 = []

query = L_genePos[0]

print (query)

query_start = int(query[2])

query_end = int(query[3])

L1.append(query)

for it in L_genePos[1:]:

#print (sbjct)

sbjct = it

#print (sbjct)

sbjct_start = int(sbjct[2])

sbjct_end = int(sbjct[3])

dist = sbjct_start-query_end

print ("=======",query, sbjct, dist, cluster_dist)

if dist > cluster_dist:

#L1.insert(0,query)

L_cluster.append(L1)

"find_geneCluster.py" [readonly] 138L, 5263C
